# Subclass analysis of donor HLA‐specific IgG in antibody‐incompatible renal transplantation reveals a significant association of IgG_4_ with rejection and graft failure

**DOI:** 10.1111/tri.12648

**Published:** 2015-09-01

**Authors:** Natasha Khovanova, Sunil Daga, Torgyn Shaikhina, Nithya Krishnan, James Jones, Daniel Zehnder, Daniel Mitchell, Robert Higgins, David Briggs, David Lowe

**Affiliations:** ^1^ School of EngineeringUniversity of WarwickCoventryUK; ^2^ Clinical Sciences Research LaboratoriesUniversity of WarwickCoventryUK; ^3^ Renal UnitUniversity Hospital Coventry and WarwickshireCoventryUK; ^4^ Department of Histocompatibility and ImmunogeneticsRoyal Liverpool University HospitalLiverpoolUK; ^5^ Department of Histocompatibility and ImmunogeneticsNHS Blood and TransplantBirminghamUK

**Keywords:** antibody, IgG_4_, multivariate analysis, rejection, renal transplantation

## Abstract

Donor HLA‐specific antibodies (DSAs) can cause rejection and graft loss after renal transplantation, but their levels measured by the current assays are not fully predictive of outcomes. We investigated whether IgG subclasses of DSA were associated with early rejection and graft failure. DSA levels were determined pretreatment, at the day of peak pan‐IgG level and at 30 days post‐transplantation in eighty HLA antibody‐incompatible kidney transplant recipients using a modified microbead assay. Pretreatment IgG_4_ levels were predictive of acute antibody‐mediated rejection (*P *=* *0.003) in the first 30 days post‐transplant. Pre‐treatment presence of IgG_4_ DSA (*P *=* *0.008) and day 30 IgG_3_ DSA (*P *=* *0.03) was associated with poor graft survival. Multivariate regression analysis showed that in addition to pan‐IgG levels, total IgG_4_ levels were an independent risk factor for early rejection when measured pretreatment, and the presence of pretreatment IgG_4_ DSA was also an independent risk factor for graft failure. Pretreatment IgG_4_ DSA levels correlated independently with higher risk of early rejection episodes and medium‐term death‐censored graft survival. Thus, pretreatment IgG_4_ DSA may be used as a biomarker to predict and risk stratify cases with higher levels of pan‐IgG DSA in HLA antibody‐incompatible transplantation. Further investigations are needed to confirm our results.

## Introduction

Renal transplantation is limited by a shortage of organs, but does offer the best quality of life and added life years for those with end‐stage renal failure. Patients previously exposed to non‐self HLA through transplant, blood transfusions or pregnancy may develop antibodies reactive to HLA 1, 2, 3. Current NHS Blood and Transplant data indicate that 47% of patients waiting for deceased donor transplant in the UK are sensitized to HLA [>10% calculated reactivity frequency (cRF)] and 25% have cRF of >85%. In the UK alone, about 200 living donor renal transplants per year have work‐up delayed or stopped because of donor‐specific HLA antibodies (DSAs). HLA antibodies are considered to have a major impact on short‐ and long‐term graft survival in those receiving transplants 4.

Among the isotypes of HLA‐specific immunoglobulin (Ig), IgG is considered to be the principal agent of humoral rejection with complement activation being the effector mechanism. IgM antibody is produced as the initial response to antigen; however, the importance of IgM HLA antibodies in renal transplant outcomes is uncertain 5, 6, 7. As the response develops, class switching may occur to IgG. There are four subclasses of IgG, and there may be progressive subclass switching from IgG_3_ to IgG_1_ to IgG_2_ and, finally, after prolonged immunization, to IgG_4_ 8, 9, 10. IgG subclasses exhibit functional differences such as complement activation and Fc receptor binding. IgG_1_ and IgG_3_ are the most effective at complement activation. Interestingly, although IgG_3_ has the greater binding efficiency to complement component C1q, IgG_1_ is the subclass most effective at complement‐dependent cell lysis 8. IgG_2_ can fix complement weakly, but IgG_4_ not at all.

There are few studies investigating IgG subclass distribution in HLA‐specific antibodies in transplantation and none considering individual specificities or looking at specific responses. From the limited number of relevant studies, it seems that in HLA‐specific sera (identified by lymphocyte binding), IgG_1_ predominates 11, 12, 13. One recent study suggest that IgG_2_ and IgG_4_ DSA do not correlate with antibody‐mediated rejection (AMR) 14.

The aims of this study were to examine the role and distribution of donor HLA‐specific IgG subclasses in the pre‐ and early post‐transplant period following HLA antibody‐incompatible (HLAi) renal transplantation using a modified Luminex microbead method and to determine any association with acute AMR and medium‐term graft survival.

## Method and materials

### Patients

Eighty patients who received HLAi renal transplants between June 2003 and October 2012 at University Hospital Coventry and Warwickshire were included in the study. Ethical approval was obtained from the local ethics committee (CREC‐055/01/03 and 13/WM/0090), and written informed consent was obtained from patients for this study. Recipients underwent flow cytometry (FC) and complement‐dependent cytotoxic (CDC) crossmatching prior to transplantation, and prospective measurement of HLA‐specific antibodies using microbead assays was made pre‐ and post‐transplant.

Prior to transplantation, patients were treated with sessions of double‐filtration plasmapheresis (DFPP) with the aim of achieving FC crossmatch negative at the time of surgery, or as low as possible after 5–7 sessions of DFPP in those with high DSA levels. In some cases, the transplant was performed in the presence of a FC‐positive or CDC‐positive crossmatch.

Immunosuppression consisted of mycophenolate mofetil 1000 mg twice daily beginning 10 days prior to transplant with dose reduction if white cell count fell below 4.0 × 10^9^/l. Tacrolimus was commenced 4 days prior to transplant for recipients of living donors, 0.15 mg/kg/day with a target trough level of 10–15 mg/l. Prednisolone, 20 mg once daily, was started at the time of surgery. Methylprednisolone, 500 mg, was given during the transplant operation. Two doses of basiliximab were given on days 0 and 4.

All cases of rejection were confirmed by renal biopsy, apart from four cases where anticoagulation was given urgently and precluded a pretreatment biopsy. In three cases early in the series, plasmapheresis was an initial therapy for AMR; in each of these cases, high and/or rising DSA levels were associated with a rise in creatinine and reduced urine output in the absence of infection. One case later in the series had the same criteria for diagnosis of rejection and required emergency dialysis for sudden oliguria. In all other cases of rejection (*n* = 42), AMR was diagnosed in accordance with the most recent BANFF guidelines 15, and in these 42 cases, C4d‐positive staining was seen in 15 patients. AMR was diagnosed in the absence of C4d primarily on the basis of peritubular capillaritis and glomerulitis 16. Rejection was treated in the majority of cases with methylprednisolone and antithymocyte globulin or OKT3, with additional plasmapheresis in fourteen and IVIg in six patients. All cases were categorized as either with episode of rejection (R) or with no episode of rejection (NR) within the first 30 post‐transplant days. Similarly, cases were categorized into death‐censored graft survival (S) and failure (F).

### Crossmatching and HLA‐specific antibody detection

Cellular crossmatching was performed prior to transplant on B cells and on T cells separated from peripheral blood (living donors) and either from peripheral blood or from spleen (deceased donors). For CDC crossmatching, 2 μl serum + 1 μl cells (2 × 10^6^/ml) were incubated for 60 min at room temperature, with and without dithiothreitol (DTT). Five microlitres of complement (rabbit serum) was added and incubated for 60 min at room temperature. Cytotoxicity was visualized using acridine orange/ethidium bromide cocktail. Anti‐human globulin enhancement was not used.

Pretransplant FC crossmatching testing was performed as previously described 15, 16. Briefly, 25 μl patient serum was added to 25 μl donor cells (10 × 10^6^/ml) and incubated at 22 °C for 30 min. Cells were washed in PBS and incubated with 100 μl goat anti‐human IgG‐FITC (Sigma‐Aldrich, Saint‐Louis, MO, USA) in the dark for 15 min at 4 °C. Cells were washed again and then incubated with 100 μl mouse anti‐human CD19‐PE (Dako, Carpenteria, CA, USA) for B‐cell crossmatch or 100 μl mouse anti‐human CD3‐PE (Dako) for T‐cell crossmatch. Following 15‐min incubation in the dark at 4 °C, cells were once again resuspended in 80 μl PBS containing 2% foetal calf serum. Samples were analysed on a Becton Dickinson FacsCalibur cytometer using cellquest Software (Becton Dickinson, Franklin Lakes, NJ, USA). The readout was the ratio of the median channel fluorescence of the test sample over that for a negative control AB serum, as relative median channel fluorescence (RMF). The threshold for a positive crossmatch was set at an RMF of 4.0 for primary grafts and 2.5 for regrafts.

Pan‐IgG HLA class I‐ and class II‐specific antibodies were identified prior to transplant using microbead assays manufactured by One Lambda Inc (Canoga Park, CA, USA) and analysed on the Luminex platform (XMap 200) (Luminex, Austin, TX, USA). Phenotype‐coated beads were used until mid‐2007, and single‐antigen beads have been used to retest the prior samples to retrospectively confirm the specificities. A positive mean fluorescence intensity (MFI) threshold of 1000 is in use in our laboratory, and antibody specificities were called when MFI exceeded 1000 in any sample from the patients’ antibody history. Thus, some cases have pretreatment MFI values <1000 in this analysis as a consequence of natural variation in antibody reactivity. Post‐transplantation antibody monitoring was performed daily for the first 2 weeks, then three times per week for the next 2 weeks tapering down to weekly testing in line with clinical progress. During episodes of rejection, testing was performed daily.

A positive cellular crossmatch was only considered to be due to DSA if the microbead assay confirmed the presence of a DSA. Uncertainty in the interpretation of the assays was resolved with auto‐crossmatching, and/or further testing of the microbead samples using reduction of IgM antibodies with DTT, or dilutions of the serum samples.

Patient serum samples were chosen for retrospective subclass analysis at specific time points in the transplant course: pretreatment [immediately before the first plasmapheresis session (or pretransplant in those with no plasmapheresis)]; the day of peak DSA post‐transplant measured by the pan‐IgG microbead assay; and at day 30 post‐transplantation. Antibody analysis was carried out in accordance with the manufacturer's instructions as previously described, but goat anti‐human IgG_1–4_ isotype‐specific monoclonal antibodies (Southern Biotech, Birmingham, AL, USA) were used to determine the levels of IgG subclass reactivity 13. Raw MFI values were used to denote positivity. IgG subclass‐specific monoclonal antibodies were validated using Luminex microbeads coated with IgG subclass‐specific human myeloma antibodies. Cross‐reactivity between isotypes was observed at <5% 13. Positive threshold MFI levels for each HLA‐specific IgG subclass were five times greater than the negative control bead incorporated into the assay: 120.6 (IgG_1_), 72.0 (IgG_2_), 62.7 (IgG_3_) and 17.2 (IgG_4_).

### Statistical analysis

HLA‐specific IgG subclasses were analysed as both the presence/absence (categorical data based on the above cut‐off values) and as MFI values (continuous data). For comparison of patients’ baseline characteristics between the groups, two types of tests were performed: Fisher's exact test (two‐tailed) for categorical data and Wilcoxon rank/Mann–Whitney *U*‐test (nonparametric) for continuous data. The null hypothesis of no difference between the groups was tested at the 5% level of significance, and this is presented by *P*‐values. IgG subclass level was considered positive if the MFI value was above the corresponding threshold for each IgG subclass and every individual bead. In cases with several HLA‐DSAs, the MFI values of each IgG subclass were added up (total IgGx). Kaplan–Meier survival analysis was performed using spss ^®^ ibm software (SPSS, IBM, Armonk, NY, USA). Log‐rank (Mantel–Cox) was used to test statistically significant difference between the groups.

To investigate the influence of IgG subclasses on the risk of acute AMR and graft failure allowing for the other potentially confounding variables, multivariate analysis was performed. In the case of categorical (R/NR) outcome, logistic regression models 17, 18 were developed, and likelihood ratio significance test was performed 19. The likelihood ratio significance test shows how many times it is more likely that the data are under one model than the other and therefore assesses the significance of presence of certain parameters in the model. Multivariate analysis for medium‐term survival outcome was carried out using Cox proportional hazard model 18.

## Results

### Comparison of baseline characteristics between the outcome groups

Characteristics of patients with an episode of AMR in the first 30 days (R) and without rejection (NR) are shown in Table 1. Pretreatment pan‐IgG DSA (both the highest single DSA bead and total pretreatment DSA levels if multiple DSA beads positive) was significantly associated with higher incidence of an episode of rejection in first 30 days. Similarly, death‐censored graft failure was significantly associated with younger recipient age, positive CDC crossmatch, and pretreatment pan‐IgG DSA (both the highest single DSA bead and total pretreatment DSA levels). Table 2 shows the distribution of DSA subclass profiles in the overall cohort of 80 patients. Subclass profiles are given as the presence or absence of each subclass in germ line heavy chain gene order (e.g. IgG_3_, IgG_1_, IgG_2_, IgG_4_). The most common subclass profiles observed are those characterized by the presence of IgG_1–4_ all positive, IgG_1_ only, IgG_3,1,2_ and IgG_1,2,4_.

**Table 1:** Baseline characteristics of transplant recipients

	Rejection (within first 30 days)			Graft failure (deaths excluded)		
	Yes = 46	No = 34	*P*	Yes = 15	No = 59	*P*
Age, median(range)	42.5 (18–68)	43 (22–67)	0.830	34 (22–50)	43 (18–67)	**0.003**
Male gender, *n* (%)	17 (37)	14 (41)	0.817	7 (47)	23 (39)	0.562
Preemptive Tx, *n* (%)	16 (35)	15 (44)	0.488	10 (67)	36 (61)	0.772
End‐stage renal failure ESRF duration in years, median (range)	13 (0–29)	10 (0–31)	0.596	7 (0–21)	13 (0–31)	0.133
Living donor, *n* (%)	45 (98)	30 (88)	0.157	15 (100)	56 (95)	0.578
DR mismatch (mm) positive, *n* (%)	38 (83)	27 (79)	0.777	13 (87)	47 (80)	0.724
Total number of mismatches (mm), median(range)	3 (1–5)	3 (0–6)	**0.132**	3 (2–5)	3 (0–6)	0.698
CDC positive, *n* (%)	12 (26)	7 (21)	0.607	8 (53)	10 (17)	**0.006**
Single highest pan‐IgG DSA (MFI), median (range)	6058 (869–13 345)	3492.5 (221–17 660)	**0.034**	8987 (775–13 345)	3788 (221–17 660)	**0.004**
Total MFI pan‐IgG DSA, median (range)	7797.5 (869–45 612)	5134 (306–37 084)	**0.011**	11 568 (775–45 612)	5793 (468–27 187)	**0.017**
Class I DSA, *n* (%)	13 (28)	17 (50)	0.312	4 (27)	24 (41)	0.575
Class II DSA, *n* (%)	13 (28)	9 (26)	0.092	3 (20)	17 (29)	0.572
Class I and Class II DSA, *n* (%)	24 (52)	10 (29)	0.067	8 (53)	24 (41)	0.394
Delayed graft function (DGF), *n* (%)	12 (26)	4 (12)	0.159	1 (7)	14 (24)	0.281
Rejection, *n* (%)				10 (67)	34 (58)	0.565

Significant differences between groups (*P *<* *0.05) are highlighted in bold. MFI values given are total IgG subclass DSA. For comparison of patients’ baseline characteristics between groups, two types of tests were performed: Fisher's exact test (two‐tailed) for categorical data and Wilcoxon rank/Mann–Whitney *U*‐test (nonparametric) for continuous data. Deceased patients (*n* = 6) have been excluded from the graft failure analysis.

**Table 2:** Distribution of subclass profiles at pretreatment time point in cohort of 80 patients analysed for rejection within first 30 days for graft failure (deaths excluded)

Subclass profile	Rejection (within first 30 days) *n* = 80				Graft failure (deaths excluded) within 2–11 years post‐transplant			
	Total	Yes	No	% Rej	Total	Yes	No	% F
‐1‐‐	8	4	4	50	7	0	7	0
3124	18	12	6	67	17	5	12	29
312‐	8	5	3	63	7	1	6	14
‐12‐	3	1	2	33	3	0	3	0
3‐‐‐	6	3	3	50	5	0	5	0
‐124	11	7	4	64	11	3	8	27
‐1‐4	6	5	1	83	6	3	3	50
31‐‐	2	2	0	100	2	1	1	50
3‐24	1	0	1	0	1	1	0	100
‐‐‐‐	17	7	10	41	15	1	14	7

Profile given as the presence/absence of each IgG subclass given in order of subclass switching (gene order), for example IgG_1_ only profile given as ‐1‐‐, IgG_3,1,2,4_ all positive given as 3124. Seventeen and 15 patients were negative in all subclasses for R/NR and F/S analysis correspondingly.

### Pretreatment levels and outcome

Table 3(a) demonstrates the associations between pretreatment IgG subclass presence and levels and rejection within the first 30 days. This shows, for rejection (R) versus no rejection (NR) groups, significant differences in positivity for IgG_1_ in pretreatment samples (*P = *0.01). IgG_4_ *levels* were also significantly higher in the pretreatment samples from the R group (*P *=* *0.003). There were no significant differences in distributions or levels of the other two subclasses, IgG_2_ and IgG_3_.

**Table 3:** Pretreatment (a) and post‐transplant (b, c) IgG subclass statistics grouped according to clinical outcome

	Rejection (within first 30 days)			Graft failure (deaths excluded)		
	Yes (R = 46)	No (NR = 34)	*P*	Yes (F = 15)	No (S = 59)	*P*
(a) Pretreatment						
IgG_3_						
*n* (%)	22 (50)	13 (38.2)	0.36	8 (53)	24 (42)	0.56
MFI, median (range)	255.5 (76.5–2793)	256.5 (75–1541)	0.63	521 (82–2793)	204 (75–1541)	0.24
IgG_1_						
*n* (%)	35 (79.5)	18 (53)	**0.01**	13 (86.7)	37 (65)	0.12
MFI, median (range)	2393 (162–24 589)	2340 (175–16 538)	0.69	6691 (175–24 589)	1121 (162–16 538)	**0.025**
IgG_2_						
*n* (%)	24 (54.5)	14 (41.2)	0.26	10 (66.6)	26 (45.6)	0.24
MFI, median (range)	581.7 (87–9472)	952.8 (75–5073)	0.62	1595 (102–9472)	432 (75–4819)	0.08
IgG_4_						
*n* (%)	24 (52.2)	12 (35)	0.17	12 (80)	23 (39)	**0.008**
MFI, median (range)	113 (24–6505)	30 (17.5–321)	**0.003**	53 (21–135)	35 (17–6505)	0.39
(b) Peak post‐transplant						
IgG_3_						
*n* (%)	25 (55.5)	11 (32)	0.07	8 (53)	25 (43)	0.57
MFI, median (range)	388 (65–1931)	286 (76.5–1770)	0.2	671 (72–1794)	288 (65–1931)	0.09
IgG_1_						
*n* (%)	35 (77.8)	14 (41.2)	**0.001**	11 (73.3)	35 (60)	0.55
MFI, median (range)	5128 (139–23 752)	2735 (197–16 973)	0.24	7308 (197–23 752)	3380 (139–17 112)	0.2
IgG_2_						
*n* (%)	23 (51)	12 (35)	0.18	10 (66.6)	23 (40)	0.08
MFI, median (range)	1497 (75–5776)	322.5 (885–5249)	**0.04**	1358 (75–5249)	910 (88–5776)	0.6
IgG_4_						
*n* (%)	31 (67.4)	10 (29.4)	**0.001**	10 (66.7)	28 (47.4)	0.25
MFI, median (range)	146 (19–2225)	54 (20–190)	**0.04**	244 (27–946)	73 (19–2225)	0.31
(c) 30th day post‐transplant						
IgG_3_						
*n* (%)	17 (39.5)	10 (29)	0.47	9 (60)	15 (26.8)	**0.03**
MFI, median (range)	444 (149–2552)	341.5 (112–1018)	0.44	500 (186–2552)	381 (112–826)	0.21
IgG_1_						
*n* (%)	28 (65)	14 (41.2)	**0.04**	11 (73.3)	29 (51.8)	0.16
MFI, median (range)	2420 (166–22 260)	1192 (131–12 280)	0.28	7483 (243–22 260)	1117 (131–11 353)	**0.034**
IgG_2_						
*n* (%)	20 (46.5)	12 (35.3)	0.36	9 (60)	21 (37.5)	0.15
MFI, median (range)	1182 (77–5865)	319 (109–4191)	0.12	1321 (84–4909)	641 (77–5865)	0.16
IgG_4_						
*n* (%)	24 (52.2)	10 (29.4)	0.21	11 (73.3)	21 (35.6)	**0.02**
MFI, median (range)	319 (17.5–3266)	129 (42–208)	0.07	311 (105–1898)	144 (17.5–3266)	0.28

Significant differences between groups (*P *<* *0.05) are highlighted in bold. MFI values given are total IgG subclass DSA. *P*‐values are derived from Fisher's exact test (two‐tailed) for categorical data and Wilcoxon rank/Mann–Whitney *U*‐test (nonparametric) for continuous data.

A multivariate model was developed to determine whether the pretreatment MFI of donor‐specific IgG_4_ or IgG_1_ was independently predictive of early rejection. Initially, all the parameters defining patients’ baseline characteristics were included into the multivariate analysis together with the total pretreatment levels of IgG subclasses, and both total and single highest pan‐IgG DSA levels. Deceased patients (*n* = 6) were included and the only three cases excluded from this analysis were as a consequence of missing baseline data, thus leaving 77 samples (43 cases in the R and 34 in the NR groups). The final multiple binary regression model (Table 4) contained 10 parameters. The likelihood ratio significance test showed that excluding single highest pan‐IgG DSA and IgG_4_ total MFI from the model decreased the goodness of the regression model by factors 4.3 and 7.6 correspondingly, and this was confirmed by the *P*‐values (*P* = 0.03 and 0 = 0.005 correspondingly).

**Table 4:** Likelihood ratio significance test for the logistic regression binary model for acute antibody‐mediated rejection (AMR) within first 30 days

Variable	Likelihood ratio	Degrees of freedom	Chi‐squared test *P*‐value
Gender	1.1076	1	0.30
Number of mismatches (mm)	11.905	6	0.06
HLA Class I and Class II DSA presence	1.038	1	0.31
Crossmatch status (xm)	4.087	2	0.13
Single highest pan‐IgG DSA (MFI)	**4.308**	**1**	**0.03**
IgG_3_ (total MFI)	1.798	1	0.18
IgG_1_ (total MFI)	0.382	1	0.54
IgG_2_ (total MFI)	0.803	1	0.37
IgG_4_ (total MFI)	**7.640**	**1**	**0.005**
Delayed graft function (DGF)	0.600	1	0.44
Constant	6.202	1	0.01

The model is developed for pretreatment samples. Baseline characteristics associated with acute AMR are highlighted in bold.

**Figure 1:**
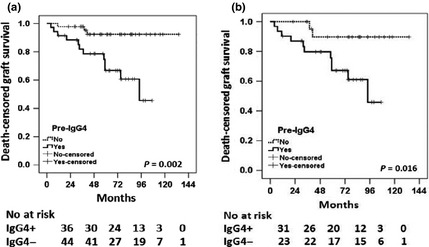
Kaplan–Meier survival analysis showing association of pretreatment IgG_4_ DSA with death‐censored graft survival for (a) overall cohort and (b) cohort with IgG_1_ DSA‐positive only cases. *P‐*value was calculated using log‐rank test. The numbers of patients at risk for each time point (in months) and in each category (IgG_4_+ and IgG_4_−) are shown underneath each figure.

The prevalence and levels of HLA‐specific IgG subclass data with respect to medium‐term outcome are also presented in Table 3(a) [in ‘graft failure (death excluded)’ column]. Of the eighty cases, there were six deaths with a functioning graft and there were fifteen graft failures during a median follow‐up period of 5 years (IQR: 3–6.7 years). This showed, for F versus S groups, significant differences in total IgG_1_ levels at pretreatment (*P *=* *0.025). The presence of IgG_4_ pretreatment was significantly higher in the F group (*P *<* *0.05). The latter was confirmed by the Kaplan–Meier method (Fig. 1a). Note that we performed the Kaplan–Meier survival analysis for all the other subclasses, and none demonstrated such associations. Additionally, given that all IgG_4_ DSA‐positive cases except one were also positive for IgG_1_, Fig. 1b shows the effect of presence of IgG_4_ in all IgG_1_ DSA‐positive patients (*P *=* *0.016) on graft survival. We have also carried out this analysis with the omission of these patients who were pan‐IgG DSA positive but negative on subclass analysis, 63 patients in total. Death‐censored graft survival analysis confirmed the original association of IgG_4_ pretreatment with poor graft outcome, with *P*‐value of 0.008.

A multivariate model was developed to determine whether the presence of donor‐specific IgG_4_ subclass, or any of the other subclasses, was independently predictive of graft failure. Seventy‐seven available cases (three excluded due to missing data, including one from the failed group) were divided into two groups according to long‐term outcome: F (*n* = 14) and S (*n* = 63). The Cox multivariate model (Table 5) showed that death‐censored graft survival was significantly worse in cases with positive pretreatment IgG_4_ DSA (hazard ratio = 5.8, *P *=* *0.035) and single highest pan‐IgG DSA pretreatment levels (hazard ratio = 89, *P *=* *0.012).

**Table 5:** Death‐censored Cox proportional hazard model for pretreatment samples

Variable	*P‐*value	Hazard ratio	95% CI for hazard ratio	
			Lower	Upper
Previous transplant	0.125	0.443	0.157	1.253
CDC crossmatch positive	0.598	1.455	0.362	5.855
Single highest pan‐IgG DSA (MFI)	**0.012**	**70.999**	**2.578**	**1955.401**
IgG_3_ (+/−)	0.464	1.694	0.414	6.932
IgG_1_ (+/−)	0.665	0.641	0.086	4.789
IgG_2_ (+/−)	0.282	0.342	0.048	2.415
IgG_4_ (+/−)	**0.035**	**5.826**	**1.129**	**30.061**
Delayed graft function (DGF)	0.165	0.225	0.027	1.853

The model is developed for pretreatment samples. Baseline characteristics associated with death‐censored graft survival are highlighted in bold.

### Dynamics of subclass levels during the first 30 days

We also analysed the trend of overall total IgG_1_ and IgG_4_ levels from peak to 30 days post‐transplantation. It was hypothesized that class switching towards IgG_4_ at day 30 post‐transplant might be associated with changes in antibody levels during the first week or two post‐transplant, rather than events which had occurred over the years pretransplant. Change in MFI levels between peak and day 30 time points was considered as rise or fall if the MFI difference was greater than 20%. For IgG_1_ levels, such rises were observed in seven cases and falls in 31 cases; for IgG_4_, there were equal numbers 20 of cases with rises and falls. This dynamic was statistically different (*P *=* *0.006). When analysed for R and NR groups separately, there was no difference in the dynamics of levels in patients for the NR group (*P *=* *0.18), but we did find a statistically significant difference in the R group (*P *=* *0.02). In the R group, IgG_1_ rose in four and fell in 24 cases compared with IgG_4_ which rose in 12 and fell in 15 cases. The MFI levels for post‐transplant samples, from which the *P*‐values were derived, can be found in Table 3(b,c).

### IgG subclass distributions at peak and day 30: associations with rejection and graft survival

We have performed univariate analysis (Table 3b,c) for peak and 30th day post‐transplant in the same way as it has been done for pretreatment levels. For R versus NR group, at peak time point (Table 3b) univariate analysis showed that the positivity of IgG_1_ and IgG_4_ was associated with rejection (*P *=* *0.001). Peak levels of IgG_2_ and IgG_4_ demonstrated a weak association (*P *=* *0.04) with rejection. At 30th day, however, there was only a single weak association (*P *=* *0.04) between IgG_1_ positivity and acute rejection (Table 3c). Multivariate regression models developed for peak and 30th day (not presented) confirmed the association of IgG_1_ at both peak and 30th day time points with rejection: the likelihood ratio significance test showed that excluding IgG_1_ total MFI from the regression model decreased the goodness of the model by factors of 8.28 for peak value and 5.39 for 30th day, and this was confirmed by the *P*‐values (*P* = 0.004 and 0 = 0.02 correspondingly). Note that although there was clear association of IgG_1_ with rejection at both post‐transplant time points, it would not have predictive value in the clinical setting. This is because the median day of onset of rejection occurred before the median day of peak DSA levels and well before day 30. These associations, however, could reflect underlying properties of the system involved in the rejection process or indeed the consequences of rejection.

**Figure 2:**
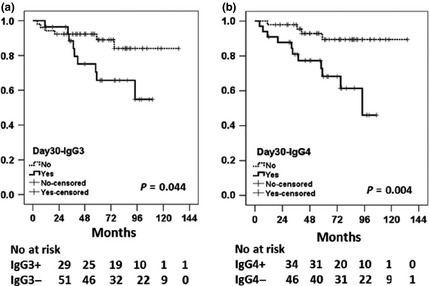
Kaplan–Meier survival analysis for 30th day post‐transplantation samples, showing association of IgG_3_ and IgG_4_ DSA subclasses with death‐censored graft survival. *P‐*values were calculated using log‐rank test for all subclasses, but only significant *P*‐values are shown. The numbers of patients at risk for each time point (in months) and in each category (IgG_3_+/IgG_3_− and IgG_4_+/IgG_4_−) are shown underneath each figure.

For medium‐term graft survival, it could be hypothesized that values at both time points could be associated with outcome, although the day 30 levels might better reflect the ongoing exposure of the graft to antibody in the medium term. For F versus S groups, the univariate analysis showed that no subclass levels at peak were associated with graft survival (Table 3b). However, for day 30, such associations are evident from Table 3(c) for IgG_3_, IgG_1_ and IgG_4_ subclasses. Table 3(c) shows for F versus S groups’ significant differences in total IgG_1_ levels at day 30 post‐transplantation (*P* = 0.034). The presence of IgG_3_ and IgG_4_ at day 30 post‐transplantation was also significantly higher in the F group (*P* < 0.05). Kaplan–Meier method (Fig. 2) confirmed the association of IgG_3_ and IgG_4_ subclasses with graft failure. However, multivariate Cox proportional hazard model developed for the 30th day (Table 6) has not revealed any significant independent associations of any subclasses with medium‐term graft failure in the presence of other patients’ characteristics at day 30.

**Table 6:** Death‐censored Cox proportional hazard model for 30th day post‐transplantation samples

Variable	*P‐*value	Hazard ratio	95% CI for hazard ratio	
			Lower	Upper
Previous transplant	0.365	0.678	0.293	1.570
CDC crossmatch positive	0.399	1.792	0.462	6.947
Single highest pan‐IgG DSA (MFI)	0.051	19.176	0.991	370.979
IgG_3_ (+/−)	0.459	2.087	0.298	14.6
IgG_1_ (+/−)	0.987	1.012	0.249	4.104
IgG_2_ (+/−)	0.292	0.281	0.026	2.982
IgG_4_ (+/−)	0.549	1.670	0.311	8.967
Delayed graft function (DGF)	0.131	0.13	0.009	1.836

## Discussion

While it is recognized that the levels of DSAs before and after transplantation correlate with the occurrence of AMR and graft failure, the predictive value of these measures is not high. Previous studies were performed using assays that measured pan‐IgG levels 20, 21, 22. Therefore, this research was performed to see whether differences in IgG subclasses would improve prediction of transplant outcome, compared to measurement of the pan‐IgG levels. DSAs of the IgG_4_ subclass were associated with increased rejection rates and reduced graft survival in univariate, multivariate and Kaplan–Meier analysis. To the best of our knowledge, this is the first demonstration of the potential prognostic value of HLA donor‐specific IgG_4_ in antibody‐incompatible transplantation. We believe this is a novel observation in transplantation and has been hitherto undetectable using the pan‐IgG assay as the relative contribution to the overall DSA response by IgG_4_ would generally be low in comparison with other subclasses.

**Figure 3:**
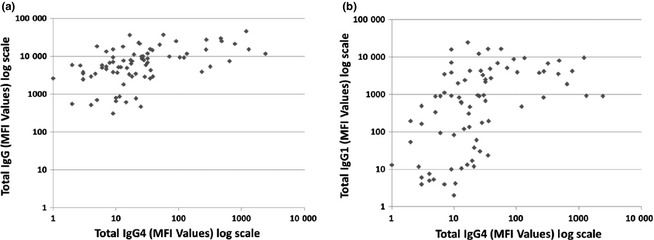
Poor correlation between (a) pretreatment pan‐IgG levels and pretreatment total IgG_4_ (*R* ^2^ = 0.1216), and (b) pretreatment total IgG_1_ and pretreatment total IgG_4_ (*R* ^2^ = 0.004).

The association of pretreatment IgG_4_ DSA with poor graft outcome was independent of IgG_1_ (Fig. 1b) and pan‐IgG DSA levels. This is strongly supported by multivariate analysis (Table 5) accounting for various confounding factors: the presence of IgG_1_ did not come up as a significant factor influencing graft outcome. Additionally, there was a poor correlation of pretreatment total IgG_4_ MFI levels with pretreatment total IgG and IgG_1_ DSA MFI levels (Fig. 3). In accordance with previously published studies by this group 13, the predominant subclass profiles found in this patient cohort include IgG_1_ only and IgG_3,1,2,4_ all positive (Table 2). Thus, in this study group, only one patient demonstrated IgG_4_ DSA in the absence of IgG_1_. Therefore, it is important that the effect of IgG_4_ DSA on graft outcome could be demonstrated even in the presence of DSA‐specific IgG_1_ (Fig. 1b).

Our assay cannot directly compare the concentrations of different IgG subclasses, but it is likely that from the known relative concentrations of IgG subclasses and the raw MFI values, in many cases IgG_4_ DSA may be in lower concentrations relative to IgG_1_ DSA. This might make the strong independent association between IgG_4_ DSA and outcomes surprising in mechanistic terms. It is possible that IgG_4_ is itself damaging to the graft and may be more potent than IgG_1_.

We analysed the outcomes in relation to both the presence and level of each subclass because there was no *a priori* reason to consider only one or the other, and we find in some cases significant associations with the presence of a subclass and in other cases significance with level. There are patterns in these apparent inconsistencies. From the data in Table 3, it can be seen that for IgG_1_, at each sample time rejection is associated with its presence, whereas graft failure associates with higher levels (for the peak level sample, this is just below statistical significance, but the trend is the same). Consistent with IgG_1_ being a potential effector of rejection and graft failure, a process could be envisaged by which its presence indicates specific immunological memory, and therefore rejection risk, while persistent or increasing high levels predispose to subsequent graft loss. The observations with IgG_4_ are difficult to explain as they are the opposite to those of IgG_1_, but they too are consistent; higher levels associate with rejection at all points (not significant at day 30, but the trend is the same), while graft failure associates with the presence of IgG_4_ in the pretransplant and day 30 samples. We have shown that the associations for IgG_1_ and IgG_4_ are independent but, until it is clear whether IgG_4_ is a direct effector in these processes or a biomarker of a process we have yet to understand, we are not in a position to try to explain this difference. It is important to stress that multivariate logistic regression analysis (Table 4) did not demonstrate any association of IgG_1_ total subclass levels with acute rejection when other confounding factors are taken into account, and of all IgG subclasses, the rejection was due to IgG_4_ increased MFI levels only.

Although generally considered anti‐inflammatory, IgG_4_ can be pathogenic in an Fc‐dependent manner 23. It is also possible that the presence of IgG_4_ indicates that there is a mature immune response in the patients, indicating a combination of antibody affinity maturation, class switching and T lymphocyte responses. Class switching is time dependent, which allows for a coordinated control of the humoral responses against persistent antigens 24, and progressive class switching from IgG_3_ to IgG_4_ is accompanied by increasing frequency of somatic VDJ point mutations and increasing affinity. This pattern of class switching has been seen in other experimental models of antibody responses against protein antigens where later stages of the response are characterized by exaggerated relative levels of specific IgG_4_ 25. Thus, where present, IgG_4_ is likely to comprise the higher affinity antibodies against HLA. These will obviously out compete IgG_1_ binding but will be limited due to lower concentration of IgG_4_. Because IgG_4_ responses required persisting antigen, IgG_4_ could also be considered a biomarker of a specific chronic T‐cell response irrespective of any direct biological effects. Such previous, chronic activation of the T‐cell compartment is a likely risk factor for rejection. The final peculiarity of IgG_4_ is that these are dynamic molecules and can exchange Fab arms, leading to the formation of a single molecule with multiple epitope binding capability which in turn may contribute to increased pathogenesis of the immune response 26, 27. A further analysis of patient samples following the transplant course beyond day 30 is indicated to determine the extent and duration of the progressive class switching.

The levels of IgG_1_‐specific DSA rose considerably from pretreatment to peak levels for the rejection group (Table 3). The trend in IgG_1_ and IgG_4_ levels from peak to 30 days post‐transplantation was significantly different in our cohort and in R group. These dynamics of the IgG_1_ response mirror the overall pan‐IgG profile we observe routinely in these cases 28. Given that IgG_1_ is the principal component of HLA‐specific IgG, this finding is compatible with other studies showing association between acute AMR and increased pan‐IgG DSA levels measured by microbead (Luminex) techniques 11, 20. Pretreatment and day 30 post‐transplant IgG_1_ levels were associated with worse graft survival. A lack of association between IgG_1_ levels at peak and longer term graft survival is not surprising, as there were large changes in donor‐specific antibody levels between peak and day 30, as we have also shown previously 20. This association is complicated by a large range of MFI levels for IgG_1_, and the dynamics of rise and fall may influence the results; further work is under investigation.

In this study, IgG_3_ was not significantly increased in prevalence or level in those who experienced early AMR, but at day 30, IgG_3_ DSA was associated with graft failure (Table 3 and Fig. 2a). This indicates similarities with recent studies suggesting an association between IgG_3_ and chronic AMR in liver transplant recipients 29, 30.

A recent study 31 described the use of a modified single‐antigen bead assay and concluded that analysis of subclass‐specific DSA prior to transplantation did not provide any substantial additional value beyond the standard pan‐IgG assay in the risk stratification of potential transplants. Although the sample size and rejection events were similar to our cohort, that study had a higher proportion of deceased donors, none with a positive CDC crossmatch, and excluded those with low total pan‐IgG levels due to assay sensitivity. Additionally, half of the cases with rejection in their cohort were late‐onset rejections and the prevalence of IgG_4_ DSA was different. The sample mix and different subclass profile may explain the differences in the results of the two studies.

Our subclass‐specific microbead assay itself is robust, with each subclass‐specific monoclonal antibody validated using a panel of carefully prepared subclass‐specific human myeloma Igs. Similar to other groups, we noticed a decreased sensitivity of the subclass‐specific assay when compared to the pan‐IgG standard assay. It should also be noted that in many cases, multiple subclass‐specific antibodies recognizing the same epitope(s) would be present in the same sera. In theory this may lead to competitive inhibition of binding to single‐antigen beads and may mean that certain subclasses with reduced affinity may have reduced binding in the assay. We have balanced this reduced sensitivity by altering our positive cut‐off threshold accordingly, with a cut‐off value of greater than five times the internal negative control bead deemed positive. This is a stringent way of determining positivity and as such provides a greater risk of assigning weakly positive samples as negative. Therefore, it may be that many of the observations here have underestimated the number of positive samples and that in turn all positive associations carry greater weight for this. The use of a ratio of reactivity (number of times greater than the negative control bead) allows us to use a ranking analysis for most of the data, which has the added advantage of eliminating the need to define a positive/negative reaction cut‐off.

A shortcoming of this study is that the C1q‐binding assay has not been performed on all the study samples, so we cannot directly compare the prognostic significance of this assay with the presence of IgG_4_ DSA, or whether any combination of assay results gives an even stronger prognostic score. However, given that IgG_4_ levels would not be expected to reflect the complement‐binding capacity of serum, as IgG_4_ does not bind C1q, it would not be expected that C1q binding would simply be correlated with IgG_4_ levels. Further studies are in progress to explore this question. This research strongly suggests that there is merit in determining the donor‐reactive IgG subclass profile prior to transplantation, as the increased presence of donor‐reactive HLA‐specific IgG_4_ appears to be predictive of early acute AMR and medium‐term graft survival independently from other variables such as crossmatch status and pan‐IgG DSA levels. In addition, the careful monitoring of IgG subclasses in the early post‐transplant period was highly informative, with a skewing of IgG subclasses towards IgG_1_ and IgG_4_, which was strongly associated with rejection. Additionally, the appearance of IgG_3_ subclass on day 30 after transplantation was also associated with graft survival, compatible with recruitment of new responses. The early identification of this shift in subclass composition may indicate clinical intervention and reduce the impact graft failure in these high‐risk transplants.

## Authorship

NK and DB: participated in research design, analysed the data and wrote the manuscript. SD: participated in clinical care, analysed the data and wrote the manuscript. TS: analysed the data. NK: participated in clinical care and wrote the manuscript. JJ: performed the research. DZ and RH: participated in clinical care, designed the research, analysed the data and wrote the manuscript. DL: performed the research, designed the research, analysed the data and wrote the manuscript.

## Funding

The work was supported by EPSRC UK (grant EP/K02504X/1) and NIHR Doctoral Research Fellowship (NIHR/DRF/2010/03/088).
